# Repurposing Drugs via Network Analysis: Opportunities for Psychiatric Disorders

**DOI:** 10.3390/pharmaceutics14071464

**Published:** 2022-07-14

**Authors:** Trang T. T. Truong, Bruna Panizzutti, Jee Hyun Kim, Ken Walder

**Affiliations:** 1 IMPACT, The Institute for Mental and Physical Health and Clinical Translation, School of Medicine, Deakin University, Geelong 3220, Australia; truongtra@deakin.edu.au (T.T.T.T.); b.panizzuttiparry@deakin.edu.au (B.P.); jee.kim@deakin.edu.au (J.H.K.); 2 Mental Health Theme, The Florey Institute of Neuroscience and Mental Health, Parkville 3010, Australia

**Keywords:** network analysis, drug repurposing, psychiatric disorders, medications, psychiatry, drug discovery, mental disorders

## Abstract

Despite advances in pharmacology and neuroscience, the path to new medications for psychiatric disorders largely remains stagnated. Drug repurposing offers a more efficient pathway compared with de novo drug discovery with lower cost and less risk. Various computational approaches have been applied to mine the vast amount of biomedical data generated over recent decades. Among these methods, network-based drug repurposing stands out as a potent tool for the comprehension of multiple domains of knowledge considering the interactions or associations of various factors. Aligned well with the poly-pharmacology paradigm shift in drug discovery, network-based approaches offer great opportunities to discover repurposing candidates for complex psychiatric disorders. In this review, we present the potential of network-based drug repurposing in psychiatry focusing on the incentives for using network-centric repurposing, major network-based repurposing strategies and data resources, applications in psychiatry and challenges of network-based drug repurposing. This review aims to provide readers with an update on network-based drug repurposing in psychiatry. We expect the repurposing approach to become a pivotal tool in the coming years to battle debilitating psychiatric disorders.

## 1. Challenges of Drug Research for Psychiatric Disorders

Psychiatric disorders are leading causes of disability, with an increasing burden and significant repercussions for health, society and the economy [1,2]. Despite some pharmacological advances, drug discovery for psychiatric disorders is particularly challenging and remains virtually stagnant. Out of 101 new drugs approved by the FDA in 2019 and 2020, only two were indicated for psychiatric disorders [3,4]. Such an outcome suggests that, compared with other diseases, drug development for psychiatric disorders has intrinsic bottlenecks that hinder the roadmap to new medications. In particular, there is a lack of understanding of the pathological mechanisms of neuropsychiatric disorders, largely due to their complex and ambiguous aetiology (genetics, environment, brain structure and function) [5,6]. Therefore, these disorders pose great challenges to the identification and characterization of biomarkers and molecular targets, as well as utilizing animal models adequately representing the disease. 

**Figure 1:**
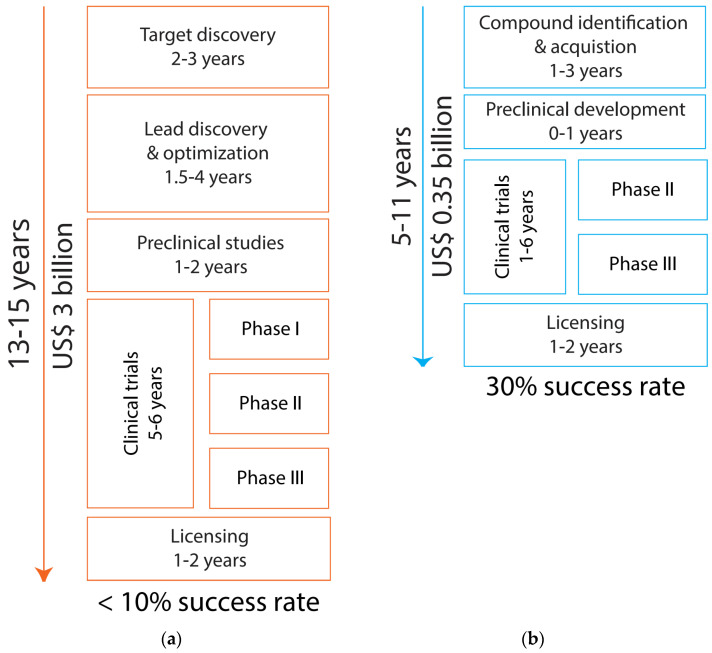
**The comparison between (a) conventional drug discovery and (b) drug repurposing.** (**a**) De novo drug discovery usually requires 13–15 years and may cost up to USD 3 billion from initial experiments to final marketing approval. Moreover, the overall success rate is only ~10%. (**b**) Drug repurposing typically bypasses several steps of the conventional approach, including not only early discovery and preclinical stages but also Phase I clinical trials. Hence, time and cost can be optimized to 5–11 years and USD 0.35 billion respectively, with an improved success rate of 30%.

Drug development is an inherently laborious, expensive, and time-consuming process, which becomes even more difficult for psychiatric disorders subserved by poorly understood mechanisms. Conventional drug discovery has long been considered a costly and risky journey (Figure 1a). The whole process usually takes approximately 13–15 years from initial discovery to final regulatory approval, and costs USD 2–3 billion [7]. The expenditure is predominated by failed candidates which are common given the low success rate of <10% [8]. 

In de novo drug discovery, a hypothesis related to the inhibition or activation of a protein/pathway would form the basis for the first step (target discovery—as shown in Figure 1a) [9]. However, psychiatric disorders are multi-faceted conditions, and it is still unknown whether targeting a key factor/pathway could lead to successful treatments [10]. The lack of experimental models not only poses further hurdles to answering that key mechanistic question but also prevents the next step of de novo drug discovery, i.e., lead discovery and optimisation (Figure 1a). This step is generally based on high-throughput compound screening or/and structure-based design but such approaches would require credible models to measure expected phenotypic traits [9]. Furthermore, novel compounds would undergo pharmacokinetics and pharmacodynamics testing including blood–brain barrier (BBB) penetration—another unique challenge of drugs targeting central nervous system (CNS) diseases such as psychiatric disorders [11].

## 2. Drug Repurposing—An Accelerated Framework for Psychiatric Drug Development

In recent years, drug repurposing or repositioning, i.e., finding new indications for drugs previously developed and/or marketed for a different disease, has become an attractive alternative to conventional drug discovery. Considering the high attrition rate of de novo drug discovery, a plethora of abandoned candidate drugs, including some that have passed safety assessment but failed due to lack of efficacy, can be recycled and utilized for new therapeutic purposes. Given the known safety profiles and bioavailability, as well as established manufacturing processes, drug repurposing can bypass some steps of conventional drug discovery and hence shorten the timeline from bench to bedside with lower cost and less risk (Figure 1b) [12,13,14]. Drug repurposing is playing an increasingly important role in the pharmaceutical industry. Out of 64 new drugs and biologics approved by the FDA in 2018, only 8 were first-in-class agents (i.e., novel drugs with a unique mechanism of action) [15]. As a shortcut to drug development, drug repurposing provides more feasible paradigms for organizations and institutions with limited resources, and potentially better financial incentives for companies to invest in rare, orphan diseases [16]. Importantly, governments and regulatory bodies are giving rigorous support including funding programs and drug repurposing public databases [17].

In the field of neuropharmacology, there have been a substantial number of repurposed drugs approved or in development. A review by Caban et al. in 2017 reported a total of 118 repurposed drugs for 203 cases in neurology and psychiatry (some drugs have been repurposed for more than one neuropsychiatric disease) [18]. Although most approved drug cases originated from the same discipline (i.e., neuropharmacology), the majority of developing cases are from outside the field [18]. For example, there are recent investigational candidates with positive results, such as tamoxifen repurposed from oncology for use as an antimanic agent (completed phase 3 clinical trials) [19], and quinidine which was repositioned from an anti-arrhythmia drug to an antipsychotic (currently entering phase 3 clinical trials) [20]. The early success of these candidates may be a glimpse of the vast untapped potential of recycling drugs from beyond the scope of neuropharmacology. 

## 3. Why Networks Matter for Psychiatric Drug Research

Across the entire process of drug repurposing (Figure 1b), the first step of compound identification is critical. Such repurposing compounds could be recognized from empirical or even serendipitous observations, with the prominent examples of valproic acid for bipolar disorder and ketamine for major depression [21,22]. While these empirical findings have earned great success in psychiatric drug research, the advent of computational techniques as well as high-throughput data from “omics” technologies have enabled us to adopt a more systematic approach to discover new therapeutic agents. These approaches also require the design of methodologies that integrate the high-dimensional but noisy data efficiently to acquire useful insights for drug discovery, leading to the application of network science in medical research. Network science is the use of multiple layers of information to identify connections among biological components that are inherently and physiologically relevant [23]. 

The fusion of network science and drug research was first conceptualized by Andrew L. Hopkins based on the premise of poly-pharmacology—one drug, multiple targets [24]. This holistic view has been appreciated in psychiatry, in which many psychotropic drugs have been shown to exhibit promiscuity as an intrinsic feature of their therapeutic effects [25]. Antipsychotics are prominent examples. Each antipsychotic drug typically targets multiple receptors and they possess distinct pharmacological profiles [5]. Hence, poly-pharmacological profiles demand consideration of multiple factors (e.g., interactions with molecular targets, downstream affected pathways) to elucidate the mechanism(s) of action of known drugs as well as to discover new therapeutic agents for psychiatric disorders [6]. Network science enables the integration of various biological elements and simultaneous consideration of their relationships in complex systems, making it a powerful system for the poly-pharmacological paradigm. 

Despite their pathological heterogeneity, psychiatric disorders have been suggested to share overlapping molecular mechanisms especially at the genetics level [26,27,28,29]. Co-morbidity is the norm rather than the exception for psychiatric disorders [30,31,32,33]. While such commonality has posed challenges to the characterisation of distinct disorders, it also offers opportunities for the utilisation of existing drugs in multiple mechanistic-related disorders [34]. Therefore, network-based approaches can leverage the interconnection between different disorders to find potential latent connections suggesting the recycling of known targets of a disorder in another disorder. 

## 4. Network-Based Drug Repurposing in Psychiatry

Previous publications have offered comprehensive reviews on network science theory [35] and capabilities in the context of medicine [36,37]. Herein, we will present major terminologies, repurposing strategies, main data resources and applications in psychiatric drug research. 

Network-based interpretation comprises three major steps from understanding to predicting and possible manipulating biological systems: (1) network inference (reconstruction of network relationships from biomedical data, mostly from high-throughput assays), (2) network analysis (harnessing the topological relationships of networks), (3) network modelling (dynamic representations of time-course perturbations of network elements under different conditions) [38,39]. Most studies so far have utilised the first two steps for static networks, but very few have advanced to dynamic network modelling [36].

**Figure 2:**
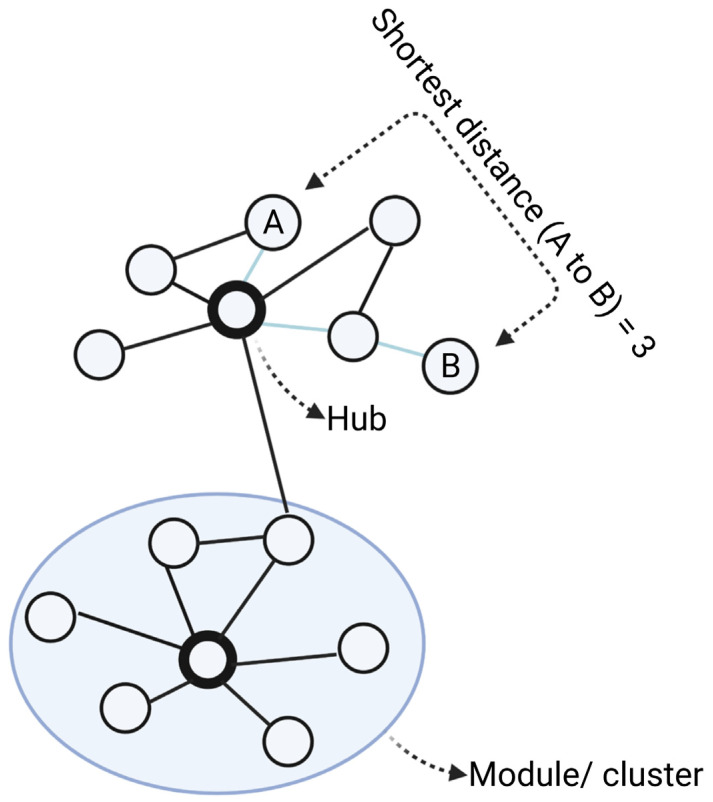
**Main elements of a network.** In the network, nodes (circles) are connected via edges (lines). For biological networks, nodes are usually biological entities (genes, proteins) and edges denote their relationships (interaction, association, similarity). From the networks, modules are clusters of closely connected nodes. Degree is the number of direct connections a node has to other nodes. Hubs are nodes with the highest degrees in the networks, meaning they have the highest number of connections. The shortest distance between node A and B is the path with the minimum number of edges from A to B. Created with BioRender.com (accessed on 2 June 2022).

A network inference approach involves “simplifying” complex systems by describing them as a map of nodes connected by edges denoting their relationships or interactions [40] (Figure 2). While networks can represent a wide range of biological processes, in the context of drug discovery research, nodes are generally molecular targets (genes, proteins), compounds (drugs) or diseases, with their relationships inferred from structural interactions (e.g., protein–protein interactions), correlation (e.g., co-expression networks) or conditional dependences (e.g., Bayesian networks) [41]. Many real-world networks including biological networks, tend to exhibit scale-free properties, which means only a minority of nodes have a greater number of neighbours than average (“hubs”), while most nodes only have a few connections [42,43,44]. Selective targeting of hubs can therefore cause much greater impact on the function of the networks than those modulations on peripheral nodes, making hubs ideal drug targets [45].

**Figure 3:**
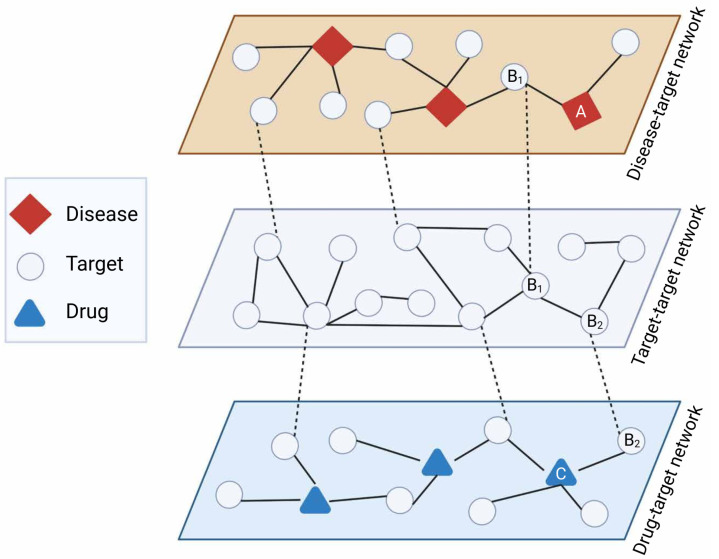
**ABC model for network-based drug repurposing.** Latent repurposing relationships can be inferred from multiple layers of network-based knowledge such as disease-target (diseasome), target–target (e.g., protein interactome), and drug–target interactions. As an example, disease A has target B_1_ exhibiting direct interaction with target B_2_ which in turn is targeted by drug C, suggesting drug C might be relevant for disease A (A → B_1_ → B_2_ → C). Created with BioRender.com (accessed on 2 June 2022).

Network-based drug repurposing efforts are generally based on Swanson’s ABC model to retrieve unknown latent knowledge from multiple sources of data incorporated in the networks [46]. An assumption of this approach is that when term A is connected to term B, and term B is connected to term C, we can assume that terms A and C are also connected. For example, an indirect link between drug and disease can be inferred from a direct drug-target connection and a direct target-disease connection. In the ABC model, A and C must originate from different domains to yield new knowledge, and B can include multiple steps to abridge from A to C (A → B_1_ → B_2_ … B_n_ → C) [47,48] (Figure 3).

**Figure 4:**
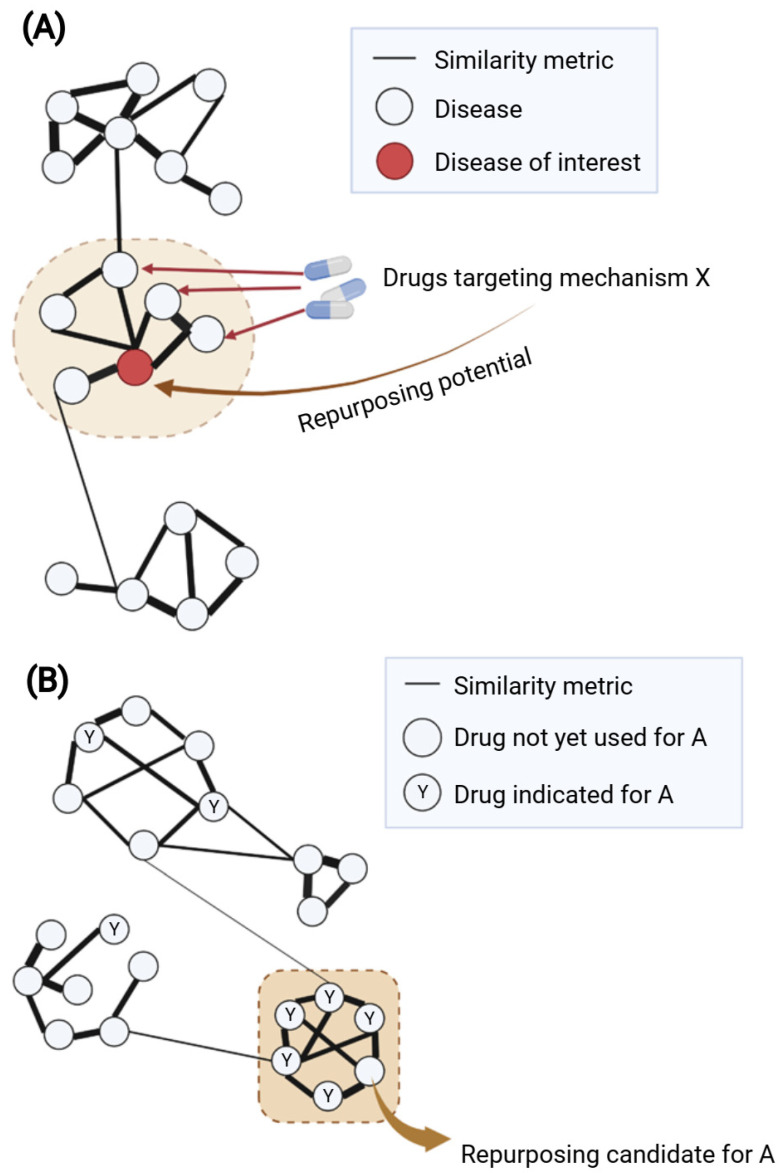
**Guilt-by-association for network-based drug repurposing using (A) disease–disease or (B) drug–drug similarity.** (**A**) Disease–disease similarity is generally inferred from one or several disease-related properties such as overlapping disease genes, symptoms or comorbidities. A weighted disease network (diseasome) can be built based on the similarity metric; herein, modules of similar nodes (diseases) can be identified. The module containing the disease of interest (highlighted in the brown dashed circle) might suggest potential shared mechanism(s) for repurposing drugs. Within this module, if multiple connected diseases have known drugs with similar mechanism X, such drugs might be repurposed for the disease of interest. (**B**) Drug–drug similarity can be calculated based on one or several properties such as chemical structures, targets, side effects or transcriptional profiles. Using the similarity metric as the weight of edges for network construction, ones can identify modules of highly similar nodes (drugs) suggesting similar mechanisms of action. When considering in the context of a certain disease A, it would be of interest to focus on the module containing multiple known drugs for disease A (highlighted as brown dashed square). Within such a module, a drug that has yet to be used for disease A might be a potential repurposing candidate due to its high similarity with other drugs used for disease A. Created with BioRender.com (accessed on 2 June 2022).

Another common approach is “guilt-by-association” (GBA), which uses similarity measures to suggest new disease indications for drugs [49]. There are two main assumptions of GBA: (1) if two diseases share a significant number of characteristics (e.g., indications, medical descriptions, mechanisms), a drug known to treat one of them may also treat the other (Figure 4A); and (2) if a drug with unknown indications and another drug with known indications share similar properties (e.g., chemical structures, transcriptional effects), they may have the same indication profile (Figure 4B). The major challenge of this approach would be how to define the robust similarity metric between drugs or diseases that concurs with similarity in mechanisms of action. 

**Figure 5:**
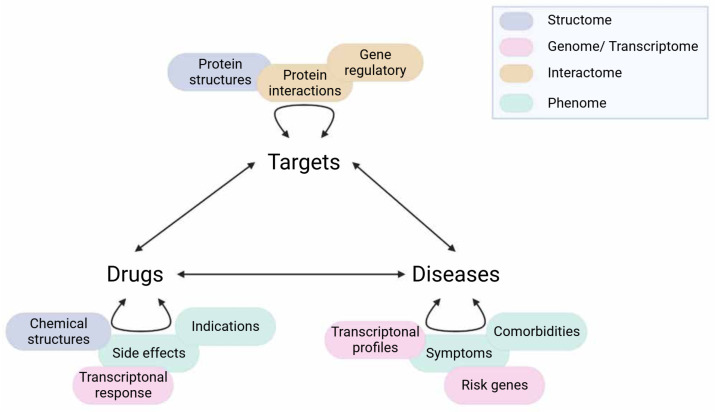
**Different data sources for network-based drug repurposing.** Curved arrows represent the associations of entities within one type (e.g., drug–drug). Multiple data sources (coloured correspondingly to their main domains such as transcriptome) can be applied to infer these associations, usually for the creation of similarity or interacting networks. Straight arrows represent the relationships between entities of different types (e.g., drug–target). For drug repurposing, the aim generally is to find a latent drug–disease connection, which can be achieved by taking the inference route from Drugs–Targets–Diseases (and vice- versa) as in the ABC model, or via Diseases–Diseases–Drugs (or Drugs–Drugs–Diseases) as in the GBA model. Created with BioRender.com (accessed on 2 June 2022).

Data for network construction can be sourced from experimental data (e.g., high throughput screening), text mining or databases (e.g., phenotypic profiles, protein interactions). Text mining is also the main strategy of literature-based drug repurposing, which shares many integrative opportunities with network-centric approaches. Hence, readers can refer to previous reviews in this domain for an in-depth methodological presentation [50,51]. The advantage of network-based approaches is the possible integration of multiple data layers to complement the incompleteness of each domain’s knowledge. Therefore, studies using network-based drug repurposing tend to utilise multiple data sources rather than one. There are various ways of data incorporation to find repurposing insights as shown in Figure 5. However, one should consider the relevance to the disease of interest (e.g., data yielded from brain tissue versus muscle tissue) and the robustness of the evidence supporting such a relationship (e.g., experimental evidence versus co-expression). Multi-omics integration has been playing a major role in the current biological interpretation and readers can refer to previous reviews of specific updates and recommendations for this approach [52]. Herein, we will focus on different types of biomedical database resources and their utility in the context of psychiatric drug discovery research (summarised in Table 1). A summary of studies using network-based drug repurposing in psychiatry is given in Table 2.

### 4.1. Structural Data (Structome)

Structural data from compounds and biological entities such as proteins and RNAs have been extensively utilized in structure-based drug repurposing [123]. The conventional structure-based approach usually requires a few predefined specific target molecules, which is not suitable for psychiatric disorders with complex pathology as mentioned in Section 3. However, network-centric approaches can incorporate the structome as a layer of information in a non-biased way to find new indications for drugs. Tan et al. used descriptions of 3D chemical structures from PubChem to calculate the similarity profiles of 965 drugs [59]. The Tanimoto-based 3D similarity scores were then combined with gene semantic similarity information and drug–target interactions to construct a drug similarity network. From this GBA approach, Tan et al. predicted new indications for 143 drugs and missing indications for 42 drugs without Anatomical Therapeutic Chemical (ATC) codes (indications not yet listed in ATC database) (Table 2). Psychotropic drugs suggested for repurposing from this study included raloxifene (from postmenopausal osteoporosis to schizophrenia) and cyclobenzaprine (from muscle spasms to sleep disorders) [59]. Raloxifene has passed a phase 4 clinical trial in participants with schizophrenia [124,125] while a phase 2 clinical trial of cyclobenzaprine was terminated prematurely due to inadequate recruitment [126]. 

### 4.2. Genome

Using the phenotype-to-genotype concept, multiple large-scale genome-wide association studies (GWAS) have identified thousands of genetic variants across the genome associated with psychiatric disorders [127,128]. Disease-associated genes located in risk loci can be inferred from GWAS data and are usually used in network analysis as a filtering layer to prioritise targets relevant to the disease. Ganapathiraju et al. used schizophrenia-associated genes in combination with protein–protein interactions to create a schizophrenia interactome [88]. Such a disease-specific network can be harnessed for target identification and testing of repurposed agents [122]. However, a major limitation of using GWAS data is the lack of directionality, making it difficult to determine whether a risk gene is up- or down-regulated in the disease phenotype. Gaspar et al. partially addressed this shortcoming via the incorporation of the GWAS summary statistics with gene expression to predict expression levels in different tissues, which were incorporated with drug–target interactions to build a bipartite tissue-specific drug–target network for major depression [73] (Table 2). 

### 4.3. Transcriptome

Among the wealth of “omics” data, transcriptomic profiling has emerged as an efficient source for computational drug repurposing due to its standardized data format, multiple comprehensive public databases, and possible implementation with network biology approaches for complex diseases [12,129,130]. The expression patterns of gene products that are connected by signalling cascades or protein complexes are expected to be more similar than those of random gene products [40,131]. With this premise, co-expression networks built upon multi-dimensional data such as transcriptomics have aided in the identification of latent mechanistic patterns of psychiatric disorders and their medications, which could be missed by conventional differential expression analysis [131,132]. 

Psychiatric disease-related transcriptional profiles, generally from post-mortem brain samples, can be readily obtained from experiments, public databases, or psychiatric-centric consortiums such as PsychENCODE and CommonMind [66,68]. The transcriptomic data can be used on its own (gene expression levels) or incorporated with GWAS data to predict genetically regulated gene expression. As an example of the former, Cabrera-Mendoza et al. used transcriptional profiles from post-mortem brain samples of substance-use disorder individuals with and without suicidal behaviour to build gene co-expression networks associated with each phenotype (Table 2). The hub genes from these networks were then subjected to drug–gene interaction testing using the DGIdb database [94] to identify drug repurposing candidates [75]. Integration of transcriptomic profiles with GWAS data was adopted by Rodriguez-López et al. for finding druggable targets in schizophrenia. The authors estimated polygenic scores based on predicted expression and associated these scores with co-expression modules to find relevant hub target genes for early intervention [74]. Gaspar et al. also applied the genetically predicted gene expression approach [73]. 

Major sources of drug-induced transcriptional profiles are generated from cell lines after treatment exposure, utilising seminal reference databases for drug responses such as Connectivity Map (CMap) [133] and the Library of Integrated Network-based Cellular Signatures (LINCS) [134]. While transcriptional profiles have been used extensively in signature-based drug repurposing for the generation and comparison of selective genes representing the phenotype of interest [129,135], their network-centric drug repurposing application is still very limited in psychiatry. An emerging systems-level approach constructing gene-regulatory networks associated with each drug treatment-cell line pair using CMap expression data can offer a comprehensive characterisation of the mechanism of action of drugs. Such a systems-level approach includes information on complex interactions between multiple entities, beyond the reductionist consideration of several signature genes [119,136]. 

The major challenge of using drug-induced gene expression in psychiatry is the lack of biological and pathological representation of the treated model systems. Transcriptional perturbations are highly context-dependent; hence, the cancerous cells used commonly in CMap and LINCS might not recapitulate the tissue-specific effects in neuronal or glial cells. The advancement in stem cell technology has propelled the generation of patient-derived induced pluripotent stem cells (iPSC), leading to the genesis of the NeuroLINCS center of omics data generation for human iPSC response in neurological diseases [137]. Since iPSCs carry the genetic information of the patients, they recapitulate the disease-related mutations that would be more representative for diseases with significant genetic factors such as psychiatric disorders [138]. 

### 4.4. Interactome

Interactomes encompass the functional interactions of biological components, which might include physical contact between proteins (protein–protein interaction networks), metabolites (metabolic networks), transcription factors and putative regulatory elements (gene regulatory networks) or functional relationships only such as phenotypic profiling networks (phenome networks) [40]. The interactome might be placed in specific biological contexts such as signalling pathways or disease-related pathways [139]. The functional interactome based on phenotypic profiles have been broadly applied for drug discovery and will be discussed separately in the context of phenome-based networks. Interactome networks tend to possess small world property: nodes are well connected with only a few paths required for the shortest distance (Figure 2). This holds highly relevant for functionally associated nodes, ensuring a quick flow of regulatory information passing between them [140]. With the premise that risk genes tend to be more connected in the network than a set of random genes, Kauppi et al. utilised the protein interactome to map drug targets of antipsychotic drugs with networks of schizophrenia risk genes (Table 2). Using network topological analysis of shortest distance, they found risk genes were significantly localised into a distinct module and overlapped with antipsychotic drug targets. Kauppi et al. then evaluated druggable risk genes without direct links to known antipsychotic drug targets to find potential novel targets for schizophrenia such as nicotinic acetylcholine receptor genes [89]. 

Given the key contribution of transcription factors in the modulation of gene expression and driving phenotypic perturbations, the transcriptional regulome has been employed by De Bastiani et al. for drug repurposing in bipolar disorders [91]. Their study inferred transcription factors–targets interactions via a reverse-engineering prediction algorithm applied on human prefrontal cortex microarray data. The transcription factor-centric network comprised of modules of gene targeted by each transcription factor, called “regulons”. Based on case-control transcriptomics data, gene set enrichment analysis (GSEA) was applied on the regulons to find enriched regulons in bipolar disorder. These regulons were used as gene expression signatures to query connectivity map for potential drug candidates reverting disease-related regulon signatures. Several compounds with known clinical relevance in bipolar disorders were identified such as antipsychotics (chlorpromazine, haloperidol) and antidepressants (maprotiline, mianserin, and desipramine). The study also found novel repurposing candidates including non-steroidal anti-inflammatory agents (meclofenamic acid, ketorolac, acetylsalicylsalicylic acid and diflorasone) and an antioxidant agent (trolox C) (Table 2) [91].

### 4.5. Phenome

The collection of phenotypic data collected from drug-induced (indications, side-effects) or disease-associated phenotypes (symptoms, disease genes) has been extensively used for drug repurposing with the availability of comprehensive public sources such as DrugBank and PharmGKB [55,93]. Zhou et al. built a drug side effect–gene system comprising two networks: drug phenotypic network of side effect profiles from SIDER [92] and protein interactome network from STRING [141]. The two networks were interconnected via drug-target associations from DrugBank [55]. Zhou et al. then applied this phenome-driven drug discovery system in finding repurposing agents for opioid use disorders. Rather than finding drugs targeting the pathological mechanism of the disorder, which is still mainly unknown, the system explored repurposing candidates sharing similar side effects or common targets with drugs causing or indicated for opioid use disorders. Using a network-based iterative algorithm, top-ranked repurposing candidates including tramadol, olanzapine, mirtazapine, bupropion and atomoxetine were identified with supporting clinical corroboration (Table 2) [116]. 

As presented in Section 3, psychiatric disorders tend to share mechanisms, such as pleiotropic genes associated with multiple disorders. By incorporating disease phenome and disease genome networks together, one can explore the common pathophysiology between diseases and infer potential reusable targets of one disease in a different disease. Such a disease-gene network was first proposed by Goh et al. as a “diseasome”—a bipartite graph including all known genetic disorders and disease genes connected by the association of genetic mutations to disorders [142]. Such a network can be interpreted for gene-gene similarity (connected if two genes share a disorder), or disease–disease similarity (linked if two disorders share a gene). While the specific application of diseasome in psychiatric disorders is still limited, Lüscher Dias et al. built a diseasome network considering multiple psychiatric and neurological disorders using text mining. They found several clusters shared by multiple disorders and their enriched functional annotations, e.g., depression with anxiety disorder (enriched for inflammatory response), bipolar disorder with schizophrenia (enriched for long-term potentiation and circadian entrainment). However, Lüscher Dias et al. did not consider common genes for their drug repurposing steps but focused on unique genes associated with each disorder as potential targets for the corresponding disorder (ABC model), shifting back to a single-disease context [118]. To our knowledge, there have been no cases using disease–disease similarity networks for drug repurposing in psychiatric disorders. An example outside of psychiatry from Langhauser et al. demonstrated how the repurposing hypothesis can be generated from a disease–disease similarity network of the diseasome, even from seemingly distinct diseases [143]. They built diseasome networks for 132 diseases based on four different relationships: shared genes, protein interactome, common symptoms and co-morbidity. From the diseasome, Langhauser et al. found the cGMP signalling pathway was associated with a cluster of disease phenotypes including neurological, cardiovascular, metabolic and respiratory diseases. This GBA approach suggested cGMP modulators as treatments for diseases belonging to this cluster. Based on this premise, the authors repurposed soluble guanylate cyclase (sGC) activators—cGMP generation facilitators—from their exclusive indications for cardiovascular diseases to neurological disorders and successfully validated their neuroprotection effects in vivo [143]. 

### 4.6. Network-Based Drug Repurposing Platforms

There are various approaches to yield network-based repurposing insights from biomedical data if one would like to build networks from the ground up, which has been comprehensively reviewed [36,37,41]. However, there are several platforms that can serve as a “one-stop shop” for network repurposing with the incorporation of multiple biological datasets, pre-constructed networks, pre-set analyses for easy access and queries of existing or user-generated data: for example, GRAND, a web-based database of gene regulatory networks specific for disease- or drug-related phenotypes inferred from prior experimental data such as protein–protein interactions, transcriptional profiles, transcriptional factor binding motifs and miRNAs predicted targets [119]. Using similarity scores based on properties of inferred regulatory networks, the CLUEreg tool of GRAND allows users to query a list of “high-targeted” and “low-targeted” genes or transcriptional factors of the disease to identify single or combinations of compounds that might “reverse” aberrant regulatory patterns [119]. Other examples of open-sourced platforms include PharmOmics and NeDRex; the former is a knowledgebase supporting gene-network-based drug repurposing and the latter allows heterogeneous network construction to mine disease modules for drug prioritization [100,120]. While these platforms would be easy to use with curated networks, users are limited by the scope of the current platforms, and how regularly they are updated. Reproducibility would be a challenge especially with commercial platforms such as IBM Watson for Drug Discovery where detailed analysing workflows are not publicly accessible [121]. Moreover, most datasets incorporated were yielded from different domains such as oncology, weakening the robustness of interpretations in psychiatry. 

## 5. Challenges of Network-Based Drug Repurposing in Psychiatry

Despite its great potential, there are major obstacles preventing network-based drug repurposing from making substantial impact: 

(1) While previous knowledge plays a major role in network construction, our current understanding of psychiatric disorders remains inadequate and biased towards well-studied mechanisms and biological entities. Even high-throughput screening data such as for protein interactions can only capture 20% of all potential interactions, leaving us an 80% incomplete interactome network with a great deal of missing gaps and fragmented clusters [144]. 

(2) Furthermore, the integration of heterogenous and high-dimensional datasets generally has to deal with disparate, incompatible or missing information [145]. To merge multiple datasets into a homogenous network would compromise accuracy due to the disregarding of biological and experimental variations affiliated with each dataset [146]. 

(3) Regardless of the scale of the network and data integrated, network representation in drug repurposing so far has only recapitulated static snapshots of the biological systems despite their dynamic nature. However, dynamic network modelling is still a major challenge due to the limited knowledge of interaction kinetics [147]. 

(4) Whilst phenotypic profiles are important data for network-based drug repurposing, similar phenotypes are not necessarily the result of similar modes of action. Genes, medication histories, and traits all play a significant role in the phenotypic outcomes of a drug’s mode of action [148]. 

(5) Repurposing candidates have been implied from various network-based approaches, yet the preclinical validation of these candidates is limited. Even though biological follow-ups are the gold-standard, the lack of representative experimental models for psychiatric disorders has posed a great obstacle to in vitro and in vivo validation of drug efficacy [6]. Most studies in psychiatry resorted to in silico validation such as literature cross-referencing, domain expert consultation and electronic health records (EHR) [149]. The literature-based validation is undertaken by mining clinical trials or PubMed articles to find supportive evidence such as the work of Lüscher Dias et al. [118]. Expert consultation is employed for a more credible evaluation of results and literature support, as done by Tan et al. [59]. While these validations are dependent on the inference of prior knowledge, the EHR-based validation can provide a more observational corroboration based on real-world clinical data. Zhou et al. employed EHR of nearly 73 million patients provided by the IBM Watson Health platform to validate repurposing candidates for opioid use disorders (OUD), using the odds of OUD remission as the outcome measure [116]. To validate repurposing drug X, they identified a cohort of OUD patients diagnosed with repurposing drug X’s original indication (disease A). This group was then split into an exposure group (patients with OUD, disease A, using drug X) and a comparison group (patients with OUD, disease A, not using drug X). The odds ratios of remission rates between these groups were then measured. They reported patient cohorts using top-ranked repurposing candidates had higher odds of OUD remission than corresponding groups without these drugs, supporting their repurposing potential for OUD [116]. A list of EHR resources can be referred from the collection of Observational Medical Outcomes Partnership (OMOP) Common Data Model (CDM) compliance databases [150]. Most of this list are commercial and private databases whose utility is mostly hampered by the restrictive access policies. However, recent initiatives such as “All of Us” have been collecting large-scale EHR data and making data widely available for approved researchers, offering valuable resources for biomedical research [151].

## 6. Conclusions and Future Perspectives

Drug repurposing has emerged as a promising alternative for de novo drug discovery and has become a vital shift in the pharmaceutical industry. Taking advantage of the expanding accumulation of biomedical data, various computational drug repurposing approaches have been facilitating informed decisions for drug research. Among those, network-based approaches offer a unique opportunity to integrate various domains of biological knowledge to discover latent repurposing candidates for complex diseases such as psychiatric disorders. Given the virtually stagnant progress of drug discovery in psychiatry, we have presented the incentives for using network-based drug repurposing for psychiatric disorders: the efficiency of repurposing drugs with verified safety records and the compatibility of network science with the poly-pharmacology concept for complex disorders. We then summarised major concepts and main strategies for network-based drug repurposing, including the ABC model and GBA approaches. Data sources and current repurposing applications for psychiatric disorders were then summarised to offer readers an update with the progress of this approach in psychiatry. However, no methodology is without limitations; thus, we presented common challenges of using network-centric approaches for drug repurposing—mostly with the noisiness and insufficiency of data resources, lack of appropriate models for follow-up validation and the dynamic representation of complex systems. 

Nevertheless, network-based repurposing holds great potential for expanding the knowledge of drug research, especially for complex disorders. Emerging techniques and resources will complement its capabilities for psychiatric research. Neuroimaging techniques such as functional magnetic resonance imaging (fMRI) offer the detection of the drug-induced perturbations of brain activity for predicting the efficacy of drug action [152]. A library of drug-related fMRI patterns might offer biomarker refences to compare the similarity between repurposing drugs with existing ones [153,154]. Its unique ability of non-evasively capturing functional differences at the brain systems level would be beneficial for psychiatric drug research given the complex nature of these diseases and inadequate experimental models. However, it is still an open challenge to incorporate the human connectome, i.e., the map of neural connections mapped via brain imaging, into the network-based drug repurposing given most biological data resources were measured at the molecular level. The emerging application of more pathological-representative preclinical models for psychiatric disorders such as iPSCs and organoids is also expected to provide more phenotypic-relevant datasets for drug repurposing and validation. A patient-derived stem cells library of drug response specifically for psychiatric disorders would offer a more accurate context-specific overview of drug action and therefore improve the robustness of network-based drug repurposing. 

To address the incompleteness of data, computational approaches are being developed for the integration of multi-dimensional data with differences in statistical properties and biological objectives. It is challenging to represent relationships between multitudinous omics data solely with traditional linear modelling. Therefore, multi-omics tools employing multivariate statistics, machine learning (ML) and deep learning (DL) approaches have been proposed to extract and predict complex non-linear patterns [52,155]. While much development and optimization are needed to generalize ML/DL models for systems-level capture of dynamics and kinetics underlying phenotypes, ML/DL has been aiding network inference and improving network coverage via the prediction of missing connections with supervised and unsupervised analyses [52,156]. While data integration is a cornerstone of network-based inference, most aggregation results in a single network endeavoring to represent a population with a broad spectrum of phenotypic differences. Despite being informative in terms of finding shared characteristics of the inspected population, aggregated networks generally ignore population heterogeneity. Emerging attention for precision medicine has facilitated the development of personalized characterization of biological perturbations. Several efforts have been made in network medicine to account for individual-level estimations, e.g., via overlaying the sample-specific expression data on the known biological networks, or interpolation of aggregated networks with and without a sample to estimate network contribution of such sample [157,158]. 

Empowered by the ever-growing amount of biomedical data and new computational analyses, the network-centric approach will keep proving itself as a powerful tool for the comprehension of vast knowledge to shed light on new repurposing candidates for psychiatric disorders.

## Figures and Tables

**Table 1:** **Summary of major data sources and their usage examples in psychiatry**.

Type of Data	Description and Resource	Examples in Psychiatry
Structome	**Chemical structures:** ChemBL [53] ChemSpider [54] DrugBank [55] PubChem [56] **Macromolecular structures:** Protein Data Bank [57] AlphaFold Protein Structure Database [58]	Schizophrenia, sleep disorder [59]
Genome/Transcriptome	**GWAS (general):** GWAS ATLAS [60] NCBI Database of Genotypes and Phenotypes (dbGaP) [61] **GWAS (psychiatry):** NIMH Repository and Genomics Resource (NRGR) [62] Psychiatric Genomics Consortium (PGC) [63] Autism Sequencing Consortium (ASC) [64] Whole-Genome Sequencing Consortium for Psychiatric Disorders (WGSPD) [65] **Human brain resources:** PsychENCODE [66] Brain Somatic Mosaicism Network [67] CommonMind Consortium [68] Allen Brain Atlas [69] **Drug response:** Connectivity Map (CMap) [70] Library of Integrated Network-Based Cellular Signatures (LINCS) [71] Drug Gene Budger (DGB) [72]	Depression [73] Schizophrenia [74] Substance use disorder [75] Autism spectrum disorder [76]
Interactome	**Protein–protein interaction:** Search tool for retrieval of interacting genes/proteins (STRING) [77] Human Protein Reference Database (HPRD) [78] **Pathways:** Reactome [79] Kyoto Encyclopedia of Genes and Genomes (KEGG) [80] **Regulome:** The Human Transcription Factors [81] RegulomeDB [82] Catalog of inferred sequence binding preferences [83] JASPAR [84] UniPROBE [85] TRANSFAC [86] **Multiple collections:** OmniPath [87]	Schizophrenia [88,89] Bipolar disorder [90,91]
Phenome	**Side effects:** SIDER [92] **Drug targets:** DrugBank [55] PharmGKB [93] Drug–Gene Interaction Database (DGIdb) [94] DrugCentral [95] canSARblack [96] KEGG DRUG [97] IUPHAR/BPS Guide to PHARMACOLOGY (GtoPdb) [98] Search Tool for Interacting Chemicals (STITCH) [99,100] Therapeutic Target Database (TTD) [101] Drug Signatures Database (DSigDB) [102] Pharos [103] **Binding assay profiles:** Psychoactive Drug Screening Program (PDSP) [104] BindingDB [105] **Disease-associated targets:** Online Mendelian Inheritance in Man (OMIM) [106] ClinVar [107] MalaCards [108] DisGeNET [109] Human Phenotype Ontology (HPO) [110] Monarch [111] GPCards [112] **Disease symptoms:** Human symptoms–disease network [113] Human Phenotype Ontology (HPO) [110] DMPatternUMLS [114] **Clinical trials:** ClinicalTrials.gov [115]	Opioid use disorders [116] Schizophrenia [117] Schizophrenia, bipolar disorder, autism spectrum disorder [118]
Network-based drug discovery platforms	GRAND [119] PharmOmics [100] NeDRex [120] IBM Watson for Drug Discovery [121]	

**Table 2:** **Summary of studies using network-based drug repurposing for psychiatric disorders.** Abbreviations: ABC: ABC model; ASD: autism spectrum disorder; ADHD: attention-deficit/hyperactivity disorder; BD: bipolar disorder; GBA: guilt-by-association model; MDD: major depressive disorder; SCZ: schizophrenia; SUD: substance use disorder; TWAS: transcriptome-wide association study; ?: unclear mechanism.

Studies	Diseases	Databases Used	Inference Model and Network Type	Key Finding (Original Indication/ Mechanism–Repurposed Indications)	Validation
[59]	Schizophrenia Sleep disorder	DrugBank PubChem	GBA: Drug–drug similarity	Raloxifene (estrogen receptor modulator → SCZ) Cyclobenzaprine (muscle relaxant → sleep disorder)	Literature-based (clinical trials, research articles), expert consultation
[73]	Depression	DGIdb ChEMBL PDSP Pharos PubChem DSigDB	ABC: Phenotype-informed drug-target network (http://drugtargetor.com/, accessed on 2 June 2022), i.e., an integration of drug-disease associations (GWAS pathway analysis *p*-values), target-disease associations (GWAS gene-wise analysis *p*-values, genetically predicted expression z-scores), and drug-target connections	Verapamil (calcium channel blocker → MDD) Pregabalin, Gabapentin and Nitrendipine (calcium channel modulators → MDD) Brompheniramine and Chlorphenamine (antihistamines → MDD) Lasofoxifene (estrogen receptor modulator → MDD) Levonorgestrel (sex hormones → MDD) Alizapride and Mesoridazine (D2 antagonists → MDD) Quinagolide (D2 agonist → MDD)	Literature-based (clinical trials, research articles)
[118]	Schizophrenia Bipolar Disorder Autism Spectrum Disorder	PubMed DrugBank Open Targets	ABC: Literature-mined disease–gene–drug association	AC-480, Mubritinib, CP724714, Trastuzumab, Ertumaxomab, and MM-302 (Target ERBB2 gene → SZ) SLC6A9 (glycine transporter → SZ) Bitopertin and PF-03463275 (? → SZ) Levetiracetan and Brivaracetam (anticonvulsant → SZ) CEACAM5 (? → BD) Lebrikizumab and Tralokinumab (act on IL3 →ASD)	Literature-based (clinical trials and research articles)
[74]	Schizophrenia	DGIdb	ABC: Brain co-expression network + TWAS predicted expression polygenic risk scores + drug-target interactions	Zonisamide (antiepileptic/ antiparkinsonian → SZ) Bevacizumab (antineoplastic agent → SZ) Fluticasone (cortisone analogue → SZ)	Literature-based (research articles)
[75]	Substance Use Disorder	DGIdb	ABC: Disease-related co-expression networks + drug-target interactions	MAOA inhibitors (antidepressants → SUD) Dextromethorphan (cough suppressant → SUB with suicide) Eglumegad and loxapine (? → non-suicidal SUD) Clozapine and olanzapine (antypsychotics SZ → non-suicidal SUD) Modafinil (sleep disorder → SUD)	Literature-based (research articles)
[76]	Autism Spectrum Disorder	STRING DrugBank Drug Targetor CMap	ABC: Disease-related co-expression networks + drug–gene interactome Mental disease and compounds knowledge graph (MCKG) based on literature mining for validation	Baclofen (GABA agonist for pain and muscle spasms → ASD) Sulpiride (D2 receptor antagonist, for SZ and ASD, confirmatory) Estradiol (steroid sex hormone → ASD) Entinostat (HDAC inhibitor → ASD) Everolimus (seizures → ASD) Fluvoxamine, Curcumin, Calcitriol, Metronidazole, and zinc (diverse mechanisms and uses → ASD)	Literature-based (research articles)
[116]	Opioid Use Disorders	STITCH SIDER STRING DrugBank	ABC: Drug side effect + protein interactome	Tramadol (pain → OUD) Olanzapine (SZ → OUD) Mirtazapine and Bupropion (MDD→OUD) Atomoxetine (ADHD → OUD)	Literature-based (clinical trials and research articles), clinical corroboration (retrospective case-control study of top candidates in population-level EHR data)
[117]	Schizophrenia	DrugBank MATADOR PDSP Ki Database BindingDB	GBA: SZ drug target–non-SZ drug interactome	264 SZ related drugs, 39 being investigated in clinical trials (Listed in Figure 3 of the corresponding publication)	Literature-based (clinical trials and research articles)
[122] repurposing based on network built by [88]	Schizophrenia	Psychiatric Genomics Consortium (PGC) HPRD Ensembl DrugBank	ABC: Disease risk gene–drug interactome	Sargramostin, Regorafenib, Theophylline (cancer and respiratory drugs → SZ) Cromoglicic acid (asthma prophylaxis → SZ) Acetazolamide (glaucoma, mountain sickness → SZ) Cinnarizine (Motion sickness, vertigo → SZ) Alfacalcidol (targets the VDR protein → SZ) Amiloride (on clinical trial for ADHD → SZ) Antazoline (targets ubiquitination and proteasome degradation → SZ) Danazol and Miconazole (target ESR1 and NOS3 associated with Alzheimer’s Disease → SZ)	Literature-based (clinical trials and research articles)
[89]	Schizophrenia	Psychiatric Genomics Consortium (PGC) STRING DGIdb	ABC: Disease risk gene–untargeted neighbor gene interactome	19 drugs to repurpose, one major example: Galantamine (Alzheimer’s disease → SZ)	Literature-based (research articles)
[91]	Bipolar Disorder	GEO CMap (via PharmacoGx package)	GBA: Transcription factor-target association	Chlorpromazine, Lavomepromazine, Perphenazine, Zuclopenthixol, Haloperidol, Promazine (antipsychotics → BD) Maprotiline, Desipramine, Mianserin (antidepressants → BD) Diflorasone (corticosteroid → BD) Meclofenamic acid, Ketorolac, Trolox c, and Acetylsalicylsalicylic acid (antiinflamatory/antirheumatic → BD)	Literature-based (research articles)

## Data Availability

Data sharing not applicable.
